# Moisture Damage Modeling in Lime and Chemically Modified Asphalt at Nanolevel Using Ensemble Computational Intelligence

**DOI:** 10.1155/2018/7525789

**Published:** 2018-04-18

**Authors:** M. R. Hassan, A. Al. Mamun, M. I. Hossain, M. Arifuzzaman

**Affiliations:** ^1^King Fahd University of Petroleum & Minerals, Dhahran, Saudi Arabia; ^2^University of Bahrain, Zallaq, Bahrain

## Abstract

This paper measures the adhesion/cohesion force among asphalt molecules at nanoscale level using an Atomic Force Microscopy (AFM) and models the moisture damage by applying state-of-the-art Computational Intelligence (CI) techniques (e.g., artificial neural network (ANN), support vector regression (SVR), and an Adaptive Neuro Fuzzy Inference System (ANFIS)). Various combinations of lime and chemicals as well as dry and wet environments are used to produce different asphalt samples. The parameters that were varied to generate different asphalt samples and measure the corresponding adhesion/cohesion forces are percentage of antistripping agents (e.g., Lime and Unichem), AFM tips *K* values, and AFM tip types. The CI methods are trained to model the adhesion/cohesion forces given the variation in values of the above parameters. To achieve enhanced performance, the statistical methods such as average, weighted average, and regression of the outputs generated by the CI techniques are used. The experimental results show that, of the three individual CI methods, ANN can model moisture damage to lime- and chemically modified asphalt better than the other two CI techniques for both wet and dry conditions. Moreover, the ensemble of CI along with statistical measurement provides better accuracy than any of the individual CI techniques.

## 1. Introduction

Moisture plays a significant role in asphalt pavement failure. Many developed countries have invested billions of dollars on roads and pavements to overcome all kinds of malfunctioning. An asphalt pavement may have different types of damage such as rutting, fatigue, creep, revealing, and moisture damage. Of these the exact nature of moisture damage phenomena remains ambiguous, as traditional macroscale and microscale tests cannot explain the damage thoroughly. Although in the last few decades researchers have made significant attempts to explore moisture damage phenomena, comprehensive understanding of the reasons for moisture damage is still lacking. In this paper, the authors attempt to characterize moisture damage in lime- and chemically modified asphalt binder at nanoscale level using Atomic Force Microscopy (AFM) and apply a number of Computational Intelligence (CI) methods to analyze and model the damage. The authors also propose an ensemble of CI along with some statistical methods to better model asphalt damage.

Different types of methods have been proposed to identify moisture damage in asphalt binder and pavement at macrolevel. For example, for the AASHTO T-283 [1] specification experiments are done in dry and wet conditions. Static Immersion Tests (ASTM D 1664, AASHTO T-182), Boil Tests (ASTM D 3625), and Indirect Tensile Tests (AASHTO T-283 and ASTM D 4867) are other examples where tests are done at macrolevel [2]. The measurement of the surface force of asphalt binder at nanoscale unit using AFM has been limited to asphalt morphology only. The problems of design, construction, and use of functional structures have been extensively analyzed at nanoscale unit in recent decade [3]. Despite the fact that asphaltic materials, such as asphalt, are mainly used on a large scale as well as in large quantities for road construction, the mechanical (macroscopic) behavior of these materials still depends to a large degree on the physical properties in terms of microscale and nanoscale level [4]. Although engineers, material producers, and researchers have explored the potential for many years, the analysis of asphalt binder properties at nanoscale unit has been found to be limited in utilizations [5]. Since all the destruction (e.g., adhesion/cohesion failures) initiates from the nanoscale to the microscale, the current focus is now being placed on nanotechnological (the technology that analyzes the properties in terms of nanoscale measurement) applications in asphalt. The derived information can be useful in multiscale modeling of moisture damage in asphalt. To the knowledge of the authors, only a few studies by Berquand and Ohler [6] (accessed January 1, 2018), Tarefder and Zaman [7], Tarefder and Ahsan [8], Hassan [9], Arifuzzaman and Hassan [10], Arifuzzaman [11], and Arifuzzaman et al. [12] have tested nanoscale moisture damage using functionalized AFM tips. In the study by Arifuzzaman and Hassan [10], the researchers modeled the moisture damage in polymer-modified asphalt, while Hassan [9] modeled the moisture damage in Carbon Nanotube (CNT) modified asphalt. Tarefder and Zaman [7] studied the effect of polymer alteration due to moisture damage in asphalt using AFM without applying any artificial intelligence to detect the damaging behavior. However, Tarefder and Ahsan [8] developed an ANN model to quantify adhesion from the AFM data. Arifuzzaman [11] developed ANN model to determine the moisture damage behavior of the CNT modified asphalt binder. None of these previous studies explained or predicted moisture damage in lime- and chemically modified asphalt binder. In this paper, the authors develop an ensemble CI-statistical technique to model moisture damage in lime- and chemically modified asphalt. In this process, first a number of well-known CI techniques are applied, namely, Artificial Neural Networks (ANNs), Support Vector Regression (SVR), and an Adaptive Neuro Fuzzy Inference System (ANFIS). Initially functionalized tips were used to measure the weak intermolecular forces (e.g., adhesion/cohesion) in polymer-modified asphalt binder systems. The level of moisture damage was measured at nano-Newton scale for varying measurements of lime, antistripping agents Unichem (UC), and spring constant values (*K* values) and varying the functionalized AFM tips carboxyl (-COOH), ammin (-NH3), methyl (-CH3), among hydroxyl (-OH), and silicon nitride (Si3N4). Since no practical attempt has been made to date to match the functional groups that exist in asphalt binder, this paper attempts to analyze the effect of these functional groups on moisture damage in asphalt using the above-mentioned CI techniques. To improve the performance of the CI techniques further, a number of statistical measurements are applied to aggregate the outputs of each of the CI techniques. Therefore, the aggregated values are considered as predicted values.

The rest of this paper is structured as follows: “Background and Literature Review” section provides an overview of AFM, ANN, SVR, and ANFIS and a literature review of moisture damage modeling using these techniques. “Ensemble of CI and statistical methods” section describes the ensemble model. “Materials Description and Experimentation” section presents the description of the dataset, the experimental setup, the experimental results, and the analysis. Finally, “Conclusion” section concludes the paper.

## 2. Background and Literature Review

In this section, AFM and the three CI techniques, namely, ANN, SVR, and ANFIS, are briefly described. At the end of each subsection a discussion about how and where these methods have been applied to model asphalt damage is provided.

### 2.1. Theory of Atomic Force Microscopy (AFM)

An AFM is a tool to investigate the topographical and surface properties of several engineering materials. It is designed to evaluate various local properties, such as height, friction, and magnetism. It scans and then calculates the local properties continuously to produce an asphalt binder bonding image. All the AFM probes has a very sharp tip with usual values 3 to 6 *μ*m tall pyramid with a 15 to 40 nm end radius. AFM scan measures the deflections of the cantilever which the lever performs through the reaction of a laser beam of the cantilever [13]. The tip is positioned with high resolution by piezoelectric ceramic that expands or contracts due to the voltage gradient and helps in positioning 3D devices with high accuracy [14].

#### 2.1.1. AFM Testing

AFM is used by the chemical engineering research groups at Harvard University to evaluate adhesion and cohesion forces. The use of AFM in the chemical process is known as chemical force microscopy [15]. AFM was also used by Beach et al. [16] to find drag off process between hexadecanethiol monolayers that automatically pulled together the silicon nitride tip as cantilever and silicon wafer. Okabe et al. [17] used hydrophobic -CH3 and -COOH terminating alkane thiols functionally modified to measure adhesion forces and to image the samples. Du et al. [18] used AFM to find thin films of polymer-generated that yields strength and modulus of elasticity. Researchers have also used nonfunctionalized AFM tips to measure the rigidity of asphalt binder [19]. Pauli et al. [20] measured the surface energy of asphalt binder using AFM. Masson et al. [19] also used AFM to execute imaging of asphalt and connected the images with different chemically analyzed ingredients of bitumen.

### 2.2. Artificial Neural Network (ANN)

Artificial Neural Networks (ANNs) have shown their versatile application to optimize and forecast immensely complex, uncertain or poorly understood situations by simulating the real field scenario for the last three decades in different disciplines of civil engineering. Following the same trend, the application of ANN in transportation engineering especially in pavement engineering has also received attention (e.g., Ceylan et al. [21], Flood and Ian [22]).

An ANN resembles the biological brain by being like a parallel distributed processor with high computational power Hecht-Nielsen and Robert [23] and interconnected processing units or neurons. The parallel processor is capable of solving nonlinear relations by storing and providing data for use and the interconnection provides computational power for simultaneous data processing in the ANN. ANNs are typically organized in layers and a typical ANN has three layers: an input layer, hidden layer(s), and an output layer. The numbers of neurons at each layer are chosen based on the system dynamics and the expected accuracy. The strength of interconnection is measured by weighting the vectors of the ANN [24]. There is no strict regulation to emulate the structure of an ANN. However, researchers have made different conclusions addressing the maximum or minimum number of nodes in the hidden layers.

### 2.3. Support Vector Regression (SVR)

Support Vector Regression (SVR) is an extension of well-known machine learning tool named Support Vector Machine (SVM) which was introduced by Vapnik [25]. SVR selects the set of points in each class (support vectors) that are the nearest to the other class and through them computes a hyperplane which is called the maximum-margin hyperplane and makes SVR robust. Using nonlinear mapping, data are mapped on a high-dimensional space and linear regression is then applied to the transformed feature space [26].

A study by Chen and Su [27] has shown that the nonlinear mapping relationship between some fundamental variables and the structural response was developed using SVM and Monte Carlo (MC) theory to calculate the failure probability and reliability of pavement. The experimental results were significantly precise, efficient, and in good agreement with the field conditions. Yan et al. [28] produced a forecast of the low number of dense asphalt-aggregate mixtures using SVM and achieved good performance compared to multiple-least-squares-regression. Ke-zhen et al. [29] used SVM as the basis of an insensitive loss function to measure pavement serviceability ratings of flexible pavement and found better performance compared to the AASHO model and the ANN model. Soltani et al. [30] found that a SVM algorithm was the most effective in forecasting the stiffness of a polyethylene terephthalate-modified asphalt mixture with a better Correlation Coefficient (CC).

SVM was used to predict the mechanical behavior of hot-mix asphalt using a comprehensive dataset and was found to be comparable to the ANN and better than a multivariate regression-based model by [31]. In another study Gopalakrishnan and Kim [32] showed the effectiveness of SVM on skilled offline pavement backcalculation and found comparable performance to Multilayer Perceptrons (MLP) in most cases and better in some special situations. Maalouf et al. [33] used SVR to predict the resilient modulus (MR) of hot-mix asphalt and found superior performance to the least squares (LS) with reduced mean-squared error and improved CC.

### 2.4. Adaptive Neuro Fuzzy Inference System (ANFIS)

Jang [34] developed the Adaptive Neuro Fuzzy Inference System (ANFIS), which serves as a basis for constructing if-then rules and Fuzzy Inference Systems (FIS). ANFIS combines FIS with the concept of ANN. The FIS displays the previous information in a set of constraints by trimming down the input-output space that needs to be optimized. A backpropagation algorithm is applied to tune the parameters of the FIS in order to achieve a better performance. Here FIS reforms the structure of the adaptive networks. Different methods like backward spreading, least squares estimation, Kalman filter, or hybrid learning algorithms in combination with numerous mathematical approaches can be implemented to optimize the ANFIS parameters.

A number of studies, for example, [35–41], have used ANFIS used to predict and control different engineering systems. Likewise, there have been several successful implementation of ANFIS in solving problems of pavement engineering [12, 42]. ANFIS and ANFIS-related hybrid learning algorithms have also been used to forecast the complex modulus of ethylene vinyl acetate-modified binder Yilmaz et al. [43]. Özgan et al. [44] predicted the stiffness modulus of asphalt core samples using ANFIS. Shafabakhsh and Tanakizadeh [45] scrutinized the changes in resalient modulus (MR) of asphalt mixtures under different loading patterns using ANFIS.

## 3. Ensemble of CI and Statistical Methods

In an attempt to further enhance the prediction of binders' adhesion/cohesion force, an ensemble of ANN, SVR, and ANFIS with a number of statistical methods is developed. In the ensemble method, the predicted values produced by ANN, SVR, and ANFIS for each input sample in the training dataset are aggregated using the statistical methods to generate a final prediction. In this regard, the following three statistical methods are applied to compute the final predicted values:

1. Average
2. Weighted average
3. Linear function

### 3.1. Aggregate by Average

In this approach of the ensemble method, the authors compute the average of the outputs of the above-mentioned CI techniques and consider the average value as the ultimate prediction of adhesion/cohesion force in nano-Newton scale. The final prediction is computed following the equation:

(1) $$\begin{array}{c} {\hat{y}}_{avg}=\frac{{\hat{y}}_{ann}+{\hat{y}}_{svr}+{\hat{y}}_{anfis}}{3}, \end{array}$$

where ${\hat{y}}_{ann}$, ${\hat{y}}_{svr}$, and ${\hat{y}}_{anfis}$ are the values predicted by ANN, SVR, and ANFIS for a given input sample (i.e., input sample = 〈tiptype, lime%, unichem%〉).

### 3.2. Aggregate by Weighted Average

A weight is assigned to each of the predicted values produced by individual CI techniques. The Normalized Root Mean Square Error (NRMSE) value for the training dataset for each individual CI method is considered as the weight for the respective CI method. Therefore, there are three weights for the three CI methods. Once the weights are obtained, the following equation is used to generate the prediction using the predicted outputs of each of the CI methods for a given input data sample:

(2) $$\begin{array}{c} {\hat{y}}_{wavg}=\frac{w_{ann}\times {\hat{y}}_{ann}+w_{svr}\times {\hat{y}}_{svr}+w_{anfis}\times {\hat{y}}_{anfis}}{w_{ann}+w_{svr}+w_{anfis}}, \end{array}$$

where ${\hat{y}}_{ann}$, ${\hat{y}}_{svr}$, and ${\hat{y}}_{anfis}$ refer to (1) and *w*_ann_, *w*_svr_, and *w*_anfis_ are the NRMSE values for the training dataset of the respective CI methods. The NRMSE is computed following the equation below:

(3) $$\begin{array}{c} NRMSE=1-\frac{\left\|y_{act}-y_{pred}\right\|}{\left\|y_{act}-y_{pred}\right\|}, \end{array}$$

where *y*_act_ is the desired value of binder force that needs to be predicted and *y*_pred_ is the predicted value produced by the respective CI method given an input sample 〈tiptype, lime%, unichem%〉.

### 3.3. Aggregate by Linear Function

In this approach of ensemble method, the set of predicted values using the training dataset are mapped to the desired output values using a linear function *f*(·). The linear function that is used in the ensemble method is as follows:

(4) $$\begin{array}{c} \vec{Y_{act}}=\overset{3}{\underset{i=1}{\sum }}b_{i}\times \vec{{\hat{Y}}_{i}}. \end{array}$$

In the above equation, $\vec{{\hat{Y}}_{i}}$ represents the list of predicted values using the training dataset and the *i*th CI method; *b*_*i*_ represents the coefficients for the *i*th CI method (here the 1st CI method is considered as ANN, the 2nd CI method is SVR, and the 3rd is ANFIS).

To compute the values of *b*_*i*_, a least square estimation is applied. In this regard, if *Y*_pred_ refers to the all the predicted values of each of the CI methods for the training dataset (i.e., column one of *Y*_pred_ would consists of the predicted values using ANN for each of the data samples in training dataset; column two would consists of the same using SVR, and the third column would contain those using ANFIS) and $\vec{Y_{act}}$ is the list of actual values which are required to be predicted for the training data samples, the value of $\vec{b}$ (where, $\vec{b}=\langle b_{1},b_{2},b_{3}\rangle$) is computed using the following equation:

(5) $$\begin{array}{c} \vec{b}={\left(\;Y_{pred}^{T}Y_{pred}\;\right)}^{-1}\vec{Y_{act}}. \end{array}$$

As soon as the value of *b*_*i*_ is available, for any test data sample the prediction is computed as follows:

(6) $$\begin{array}{c} {\hat{y}}_{linear}=b_{1}\times {\hat{y}}_{ann}+b_{2}\times {\hat{y}}_{svr}+b_{3}\times {\hat{y}}_{anfis}, \end{array}$$

where ${\hat{y}}_{ann}$, ${\hat{y}}_{svr}$, and ${\hat{y}}_{anfis}$ refer to (1)

## 4. Materials Description and Experimentation

### 4.1. Materials

In this study, the aim is to model the moisture damage in lime- and chemically modified asphalt. Therefore, the authors have used the following materials to mix with the asphalt binder: lime and Unichem. Details of the additives are provided below.

#### 4.1.1. Lime

Lime has been used in the hot-mix asphalt/bitumen pavement industry for more than 25 years. It contributes to both the rheological and mechanical properties in asphalt mixtures. It also improves moisture sensitivity resistance, as well as fracture toughness strength, and reduces the rate of aging due to oxidization in asphalt binders. In this study the authors added 0.5%, 1.0%, and 1.5% lime by weight of asphalt to prepare the AFM samples for testing.

#### 4.1.2. Unichem

A dark brown colored antistripping agent, Unichem, is liquid and has a viscosity of 236 mPa·s at 37.8°C, a density of 8.31 lb/gal, and an ash point of 100°C. Heat stable ingredients in Unichem help it to perform efficiently in verifying heating condition. Unichem has a good capacity to be absorbed to increase the wettability of the aggregate surface, and this plays a significant role in coating the aggregate easily. As an antistripping agent, unlike lime, Unichem does not modify asphalt chemically. It may be effective in concentrations ranging from 0.25% to 1.5% by weight of the asphalt binder. Therefore, in the experiments, the authors used Unichem concentrations within the range of 0.25% to 1.5% with respect to the weight of the asphalt binder.

#### 4.1.3. AFM Testing and Tips Functionalization

Functionalization of AFM tips was carried out to simplify the asphalt binder chemistry through various groupings like -COOH, -NH3, -CH3, and -OH. Asphalt binder has hydrogen atom-saturated long carbon chains. It has single C-C and C-H bonds with evenly distributed electrons which are slightly inclined to move around. According to Little and Jones [46], these long carbon chains saturated with hydrogen are considered nonpolar in nature. The Van der Waals forces, which play a significant role due to their additive nature in these large molecules, facilitate the interaction of these nonpolar molecules. Asphalt binder consists of some hetero atoms, such as sulfur (S), nitrogen (N), and oxygen (O), which form different functional groups in the asphalt system. A group of atoms with a particular arrangement is called a functional group. This kind of group governs a specific type of characteristics in the entire molecule. The composition of a specific group dictates the names of the functional groups. For example, -COOH is a carboxyl functional group in the asphalt molecule. Typically, the most common functional groups in asphalt binder are -COOH, -NH3, -CH3, and -OH. Therefore, these functional groups were implemented to the AFM tips in this investigation. A total of four different AFM tips are used in the current research. They are different from the different chemical view point and are modified with asphalt functionals such as COOH, -NH3, CH3, and OH. All the types were classified as either hydrophilice or hydrophobic. The COOH and OH are hydrophilic tips and the CH3 and -NH3 are the hydrophobic tips. Probing the film of asphalt binder surface with the help of functionalized tips facilitates and measures cohesion forces between the two asphalt molecules. The functionalization task was done with the help of a professional company (Novascan Technologies, Ames, IA, USA). The -COOH, -NH3, -CH3, and -OH groups which are the major part of asphalt are used to functionalized the tips.

**Figure 1:**
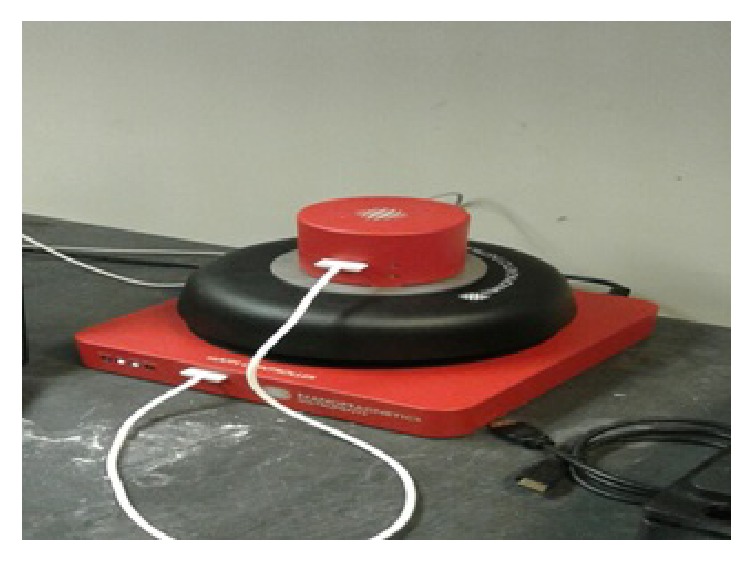
The AFM machine used in the experiments.

In AFM testing the sharp tip of the free end of its cantilever scrutinizes the surface of asphalt binder. This tip has a beam bounce cantilever with a length of 125 *μ*m with 90 kHz frequency and a spring constant (*k*) value of about 3 N/m. Here silicon nitride (Si3N4) AFM tips were functionalized with the above-mentioned functional groups from Novascan Technologies (USA). The tips were adjusted with the self-accumulation of a thin monolayer film onto AFM tips followed by immersion of the AFM tips in a solution of thiol Vaidya and Chaudhury [47]. Thiol is connected with the AFM tip surface and the competent functional group by its two ends. The attraction or repulsion generated between the tips and the binder surface results in bending or deflection of the cantilever. This deflection of the cantilever is measured by the built-in laser beam reflection facilities in AFM. Once this deflection is measured, it is converted to acting attraction or repulsion on the cantilever tips through multiplying by the spring constant. It is a consequence of the space linking the AFM tip and the sample surface.  Figure 1 shows the AFM that has been used to collect data in this paper. The main challenge in asphalt testing with AFM tips is the stickiness of asphalt binders. The sample is usually very soft at elevated temperature which causes the tips to be contaminated and even some times damaged by the asphalt. Thereby, the whole testing was conducted under noncontact mode in order to be keep the AFM tips safer from the asphalt contamination. Moreover, all the samples were put in freezer before testing so that it may not have a softer surface which is undesirable during AFM testing.

### 4.2. Asphalt Sample Preparation Using Binders

The asphalt samples were prepared in the asphalt concrete laboratory. The samples were 10 × 10 mm in length by width and thickness of about 1 mm. Such dimensions are ideal for AFM testing. The idea behind the samples thickness came from the actual asphalt coating when the aggregates are coated by the binder during the mixing time. The samples were deposited in the glass substrate which later on were inserted on the scanner for imaging as well as finding adhesion and cohesion forces. All the fresh samples went through AASHTO T-283 [1] standard procedures for the moisture damage simulation.

### 4.3. Dataset

As discussed in Section 4.1, the asphalt binder's adhesion/cohesion force varies with the variation of lime and Unichem binders. In this study, data were collected by varying the lime and Unichem as asphalt binder and then used AFM to measure the adhesion/cohesion force at nanoscale level. Since this study aims to model the moisture damage in lime- and chemically modified asphalt binder, the inputs to the CI techniques used in this study are AFM tip types and the percentages of lime and Unichem, while the predicted outputs are the adhesion/cohesion forces.  Table 1 summarizes the adhesion/cohesion forces measured for the varying values of lime and Unichem as well as the AFM tip types for two different conditions: wet and dry.

### 4.4. Moisture Damage Modeling Results

In this study one of the aims is to apply CI techniques in modeling asphalt damage due to moisture. As stated in “Background and Literature Review” section, three well-known CI methods, ANN, SVR, and ANFIS, have been used. Each of the methods was used for both wet and dry asphalt samples. Many researchers (Mas and Ahlfeld [48], Hota et al. [49]) have used 80% of the data for training their proposed computational model and the remaining 20% of data for testing the efficacy of the model; that is, these 20% data are kept unknown to the trained model. The authors of this study followed the same approach in the experiment and hence used 80% of the data (selected randomly) for training the respective CI methods and the remaining 20% data for testing the trained methods. Tables 2–5 report the experimental results in terms of the following performance metrics:

(1) Normalized Root Mean Square Error (NRMSE) (refers to (3))
(2) Correlation Coefficient (CC):
  (7) $$\begin{array}{c} CC=\frac{n\sum y_{act}y_{pred}-\sum y_{act}\sum y_{pred}}{\sqrt{n\sum y_{act}^{2}-{\left(\sum y_{act}\right)}^{2}}\sqrt{n\sum y_{pred}^{2}-{\left(\sum y_{pred}\right)}^{2}}} \end{array}$$
(3) Mean Absolute Percentage Error (MAPE):
  (8) $$\begin{array}{c} MAPE=\frac{100}{n}\overset{n}{\underset{i=1}{\sum }}\left|\;\frac{y_{act}-y_{pred}}{y_{act}}\;\right| \end{array}$$

In the above performance metrics, *y*_act_ and *y*_pred_ refer to (3) and *n* represents total number of data samples that are used to evaluate the respective CI technique. The CC value “ONE” indicates perfect correlation where “ZERO” indicates no correlation between the prediction and actual/measured outcome. The MAPE is the indication of the error as a percentage to depict it explicitly and therefore a smaller value of MAPE indicates better performance. Similar to the MAPE, a smaller NRMSE value indicates better performance.

### 4.5. Analysis of Experimental Results

Tables 2 and 3 show the moisture damage modeling performances of the three CI methods: ANN, SVR, and ANFIS. From these tables, overall, it is noticed that, for wet samples, ANN can model the asphalt damage (in terms of adhesion/cohesion force prediction), with high accuracy. For dry samples, the prediction results of the ANN model are also very satisfactory compared with the performances of the other CI methods used in this study.

In evaluating the performance of wet samples, the experimental results in Table 2 indicate that the NRMSE deviates from 0.6905 (by ANFIS for the test data) followed by SVR (NRMSE = 0.6170 for test data) and then the ANN produces an NRMSE value of 0.6017. These results prove that ANN performs better than the other two methods in modeling moisture damage to lime- and chemically modified asphalts. A similar performance trend is noticed for the performance metrics CC and MAPE for wet samples. From these findings, a conclusion can be drawn that ANN is the most preferred CI method for moisture damage modeling at nanoscale level. All these analyses indicate that the performance of ANN is convincing and its performance is very consistent for modeling moisture damage in wet samples of asphalts.

Table 3 shows the prediction performances (in terms of NRMSE) of CI techniques for dry samples. As shown in the table, ANFIS produced an NRMSE value of 0.6958 (for test data), SVR produced 0.7062 (for test data), and ANN 0.6049 (for test data). Although ANFIS performed better than SVR, the performance of ANN was the best of the three. However, the large difference between the prediction performance of training and test data for all the CI methods (e.g., SVR: 0.7062 − 0.3596 = 0.3466; ANN: 0.5859 − 0.2667 = 0.3192; and ANFIS: 0.6958 − 0.3464 = 0.3494) indicates that there may be a possibility of further improving the performances of each of the methods. Similar to the performance metric NRMSE, a large difference is seen between the CC values for training and testing data, respectively. Nonetheless, a CC value 0.8196 is still a good prediction performance due to being close to the exact performance (i.e., 1.0). An analysis of MAPE values also identified a similar performance trend to those of CC and NRMSE. Interestingly, for MAPE, the performances gap between training and test data is very small (for ANN: 0.2099 − 0.1864 = 0.0235) and therefore these performances can be considered consistent.

Having achieved a very good prediction of the adhesion/cohesion forces of lime- and chemically modified asphalts, an attempt is made to enhance the prediction performances of the CI methods; an ensemble of the CI methods with a number of statistical methods is developed, as discussed in “Ensemble of CI and Statistical Methods” section. Tables 4 and 5 show the prediction performances of the three different ensemble methods. As shown in Table 4, the performance of the ensemble of CI-average method produced a very good prediction performance in terms of NRMSE, CC, and MAPE for the wet asphalt samples. It is encouraging to achieve better accuracy than any of the individual CI methods for the wet samples (both training and test datasets) of lime- and chemically modified asphalts. The prediction accuracies of the ensemble methods of CI with average and weighted average, respectively, did not produce a better prediction accuracy than that of the ANN. However, the ensemble of CI-linear function produced an accurate prediction and was better than any prediction accuracies of ANN, SVR, and ANFIS. Once again, the better prediction performances of the ensemble methods by combining the predicted values of ANN, SVR, and ANFIS using average, weighted average, and linear function techniques, respectively, would encourage professionals to use these methods for modeling moisture damage at nanoscale.

**Figure 2:**
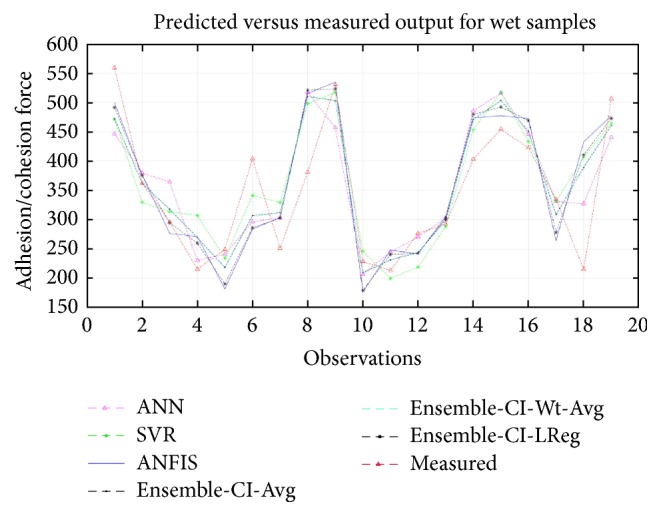
Predicted and measured adhesion/cohesion forces for wet samples.

**Figure 3:**
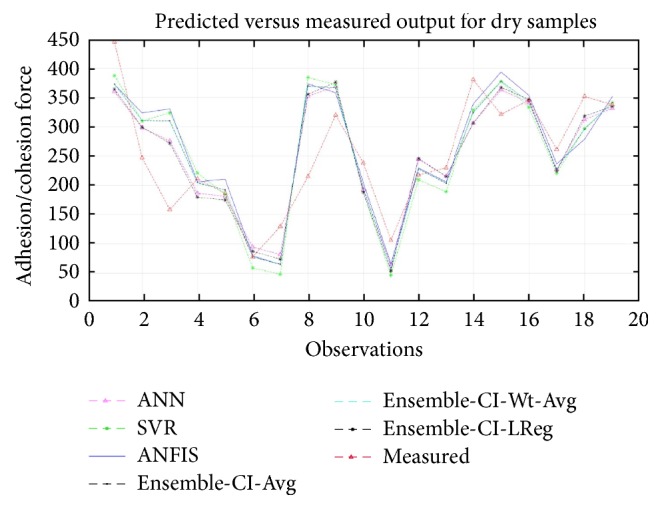
Predicted and measured adhesion/cohesion forces for dry samples.

To summarize, Figure 2 plots the measured versus predicted adhesion force data (for wet samples) produced by each of the three CI methods and the three different ensemble methods. As the plot shows, the values predicted by the ensemble CI-average technique are very close to the measured values, especially for the data samples (observations) from 2 to 5 and 9 to 13. This further suggests the superiority of the ensemble techniques to the individual CI methods in modeling moisture damage in asphalt samples under wet conditions.  Figure 3 plots the measured versus predicted values for dry samples. This plot reveals that the predicted values produced by the ensemble of CI-linear function are very close to the measured values of adhesion/cohesion forces. This further confirms that the ensemble techniques have merit for modeling moisture damage of lime- and chemically modified asphalts under dry conditions.

## 5. Conclusions

In this study, an analysis of moisture damage in lime- and chemically modified asphalts was accomplished using a number of CI techniques, namely, ANN, SVR, and ANFIS. Ensembles of CI and other statistical methods (average, weighted average, and linear function) are also developed to better model the moisture damage of asphalts under both dry and wet conditions. It is well known that binders can improve moisture damage in asphalt. In this study, the authors used lime and chemical as asphalt binders, and the adhesion/cohesion force was then computed at nanoscale using AFM. Since, it is quite difficult and expensive to do the experiments for a range of different amounts of asphalt binders and measure the adhesion/cohesion forces accordingly, computational modeling of the damage with the variations of asphalt binders was generated. The experimental results reveal that an ensemble of CI techniques following the averaging of each of the outputs of CI techniques can model asphalt damage under wet conditions, while an ensemble of CI techniques along with a linear function can produce very accurate predictions of adhesion/cohesion forces under dry conditions. In conclusion, an ensemble of CI-statistical measurements can be applied to capture the complex system of relationships among various chemical functional groups that might influence the intermolecular adhesion/cohesion forces of lime- and chemically modified asphalt due to moisture-induced damage. Our future plan is to extend this research to validate the performance of asphalt binders though comparing the adhesion/cohesion forces at nanoscale and macroscale units, respectively, that would follow the CI techniques developed in this study.

## Figures and Tables

**Table 1:** Adhesion/cohesion force summary for varying lime and chemical binders in asphalts.

Parameter	Dry samples	Wet samples
	Nano-Newton	Nano-Newton
Maximum	444.37	679.17
Minimum	31.02	115.27
Average	237.47	355.35
Standard deviation	116.24	130.82
Kurtosis	−1.09	−0.80
Skewness	−0.33	0.52

**Table 2:** Performance measurement of the ANN, SVR, and ANFIS for modeling asphalts (wet samples).

	SVR		ANN		ANFIS	
	Train	Test	Train	Test	Train	Test
NRMSE	0.4589	0.6170	0.4356	0.6017	0.3545	0.6905
CC	0.8891	0.7986	0.8988	0.7996	0.9342	0.7741
MAPE (%)	0.1579	0.1696	0.1488	0.1532	0.1763	0.2558

**Table 3:** Performance measurement of the ANN, SVR, and ANFIS for modeling asphalts (dry samples).

	SVR		ANN		ANFIS	
	Train	Test	Train	Test	Train	Test
NRMSE	0.3596	0.7062	0.2667	0.5859	0.3464	0.6958
CC	0.9323	0.7847	0.9649	0.8196	0.9372	0.7749
MAPE (%)	0.1952	0.2612	0.1623	0.2125	0.1763	0.2358

**Table 4:** Performances of the ensemble of CI-statistical methods for modeling asphalts (wet samples).

	Ensemble of CI-average		Ensemble of CI-weighted average		Ensemble of CI-linear function	
	Train	Test	Train	Test	Train	Test
NRMSE	0.3750	0.5976	0.3705	0.6003	0.3449	0.6490
CC	0.9269	0.8099	0.9288	0.8087	0.9383	0.7903
MAPE (%)	0.1352	0.1495	0.1387	0.1505	0.1205	0.1834

**Table 5:** Performances of the ensemble of CI-statistical measurements for modeling asphalts (dry samples).

	Ensemble of CI-average		Ensemble of CI-weighted average		Ensemble of CI-linear function	
	Train	Test	Train	Test	Train	Test
NRMSE	0.3057	0.6526	0.3034	0.6497	0.2656	0.5813
CC	0.9520	0.7959	0.9528	0.7969	0.9651	0.8219
MAPE (%)	0.1703	0.2222	0.1694	0.2195	0.1645	0.2112
